# Supplementary material for: Development of chemokine network inhibitors using combinatorial saturation mutagenesis
Source: Commun Biol. 2025 Apr 3;8:549. doi: 10.1038/s42003-025-07778-6 (PMC11969024; doi:10.1038/s42003-025-07778-6)
Supplement: Supplementary file 4 — Supplementary Data 2 [file 42003_2025_7778_MOESM4_ESM.zip › SupplementaryData3/scripts/xattune_ci.tar!/./script/analysis/JK/R62_CXCL11_HD2CM_plot.pdf]

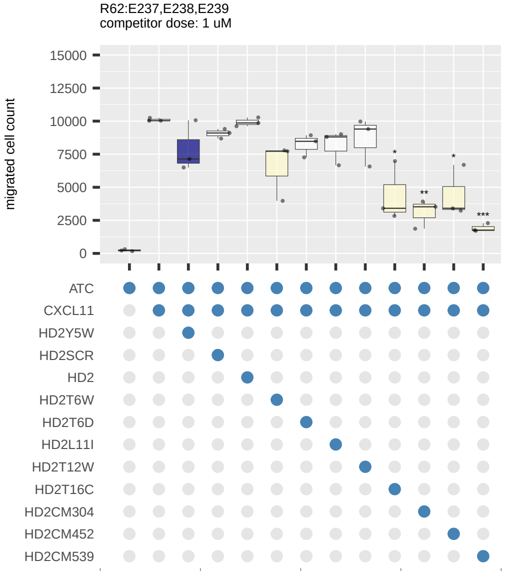
R62:E237,E238,E239
competitor dose: 1 uM
migrated cell count
15000
12500
10000
7500
5000
2500
0
*
**
*
***
ATC
CXCL11
HD2Y5W
HD2SCR
HD2
HD2T6W
HD2T6D
HD2L11I
HD2T12W
HD2T16C
HD2CM304
HD2CM452
HD2CM539
